# Evaluation of Digital Imaging Accuracy Among Three Intraoral Scanners for Full-Arch Implant Rehabilitation

**DOI:** 10.3390/diagnostics16010025

**Published:** 2025-12-21

**Authors:** Tareq Hajaj, Ioana Veja, Cristian Zaharia, Ioana Elena Lile, Mihai Rominu, Cosmin Sinescu, Florina Titihazan, Evelyn-Beatrice Toman, Andrei Bogdan Faur, George Dumitru Constantin

**Affiliations:** 1 Department of Prostheses Technology and Dental Materials, Faculty of Dentistry, Victor Babes University of Medicine and Pharmacy, 2 Eftimie Murgu Sq., 300041 Timisoara, Romania; tareq.hajaj@umft.ro (T.H.); rominu.mihai@umft.ro (M.R.); sinescu.cosmin@umft.ro (C.S.); florina.titihazan@umft.ro (F.T.); evelyn-beatrice.toman@student.umft.ro (E.-B.T.); 2 Research Center in Dental Medicine Using Conventional and Alternative Technologies, Faculty of Dental Medicine, Victor Babes University of Medicine and Pharmacy, 9 Revolutiei 1989 Ave., 300070 Timisoara, Romania; 3 Department of Dental Medicine, Faculty of Dentistry, “Vasile Goldiș” Western University of Arad, 310025 Arad, Romania; veja.ioana@uvvg.ro; 4 Department of Prosthodontics, Victor Babes University of Medicine and Pharmacy, B-dul Revolutiei 1989, No. 9, 300580 Timisoara, Romania; andrei.faur@umft.ro; 5 Discipline of Clinical Practical Skills, Department I Nursing, Faculty of Medicine, Victor Babes University of Medicine and Pharmacy, 300041 Timisoara, Romania; george.constantin@umft.ro

**Keywords:** intraoral scanners, digital impression technique, trueness, precision, reproducibility, dental implants, prosthodontics

## Abstract

**Background/Objectives:** Accurate full-arch implant impressions are essential for predictable digital prosthodontics, yet the performance of different intraoral scanners (IOSs) remains variable. This in vitro study compared the trueness and precision of three widely used IOSs-Sirona Primescan, 3Shape TRIOS Core, and Medit i700-in a standardized full-arch implant model. **Methods:** A maxillary model with six multi-unit implants was digitized using a high-accuracy laboratory scanner to obtain the reference dataset. Each IOS was used to perform ten scans, exported as unmodified STL files. Accuracy was evaluated in Geomagic Control X through a two-step alignment and a peri-implant region-of-interest deviation analysis. Trueness (mean absolute surface deviation, µm) and precision (SD) were compared using one-way ANOVA with Tukey’s test (α = 0.05). **Results:** Primescan and TRIOS Core showed comparable trueness (202.76 ± 13.89 µm and 204.21 ± 2.61 µm, respectively), while Medit i700 demonstrated significantly higher deviations (221.05 ± 6.28 µm) (*p* < 0.05). TRIOS Core exhibited the highest reproducibility across repeated scans. **Conclusions:** The three scanners demonstrated measurable accuracy differences under standardized conditions. Primescan and TRIOS Core performed similarly in trueness, with TRIOS Core achieving superior precision. Medit i700 showed higher deviation values but remained consistent in its performance. These findings highlight measurable differences in accuracy and reproducibility among intraoral scanners under standardized laboratory conditions and may assist clinicians in selecting appropriate devices for full-arch digital implant workflows; however, clinical validation is required to confirm their performance in vivo.

## 1. Introduction

The integration of digital technologies into implant dentistry has transformed diagnostic, surgical, and prosthetic workflows, with intraoral scanners (IOSs) playing a central role in modern prosthodontics. IOSs enable direct digital capture of implant positions and peri-implant soft tissues, reducing distortions associated with conventional impression materials and improving treatment predictability, laboratory communication, and patient comfort [1,2,3,4]. Osseointegrated implants remain a reliable solution for partial and complete edentulism [5], yet the long-term success of implant-supported prostheses depends strongly on impression accuracy and the passive fit of the final framework. Even minor misfits can alter load distribution, contribute to screw loosening or fracture, and accelerate marginal bone loss and peri-implant inflammation [6,7]. Scan bodies offer a standardized geometric reference that is automatically recognized in CAD systems, supporting precise framework and abutment design [8]. However, IOS accuracy can be influenced by factors such as difficulties in capturing subgingival margins, operator technique, and cumulative stitching errors over full-arch spans [9,10], while the absence of universally accepted scanning protocols further contributes to variability in reported outcomes [11].

Several systematic reviews and comparative studies have evaluated IOSs in single-implant or short-span situations, generally reporting trueness within clinically acceptable thresholds [12,13,14]. However, full-arch impressions present additional challenges due to the larger field of view and the cumulative effect of image stitching across multiple implant sites [15]. Although digital workflows have gained traction, conventional impressions remain prevalent; however, systematic reviews show that full-arch intraoral scans achieve accuracy within clinically acceptable thresholds when compared to conventional methods. In this context, discrepancies of even a few tens of microns per unit can aggregate into clinically significant misfits across the arch. While some studies have assessed selected IOS devices in vitro or in vivo, direct head-to-head comparisons of commonly used scanners under standardized full-arch conditions remain scarce [16,17,18].

Among the devices widely adopted in clinical practice, the Sirona Primescan, 3Shape TRIOS Core, and Medit i700 represent distinct technological approaches and price segments. Each has demonstrated satisfactory performance in partial arch workflows, but their relative accuracy and reproducibility in demanding full-arch, screw-retained protocols are not yet fully clarified. Addressing this knowledge gap is essential, as clinicians increasingly rely on IOSs to guide the fabrication of complex prostheses where passive fit is critical.

Intraoral scanners serve not only as impression tools but also as digital diagnostic instruments for detecting discrepancies, assessing implant alignment, and guiding treatment planning. Therefore, understanding the diagnostic accuracy of 3D datasets is essential for full-arch implant rehabilitation.

The aim of this in vitro study was therefore to compare the accuracy of these three intraoral scanners against a high-precision laboratory reference in a standardized full-arch model with six implants. Specifically, we sought to evaluate their trueness and precision in order to determine their suitability for screw-retained, implant-supported restorations and to provide clinicians with evidence-based guidance for scanner selection.

The null hypothesis was that no significant differences would be observed in trueness or precision among the three intraoral scanners when used for full-arch implant digital impressions.

## 2. Materials and Methods

### 2.1. Study Design

This primary analytical in vitro study aimed to evaluate the accuracy of intraoral scanners (IOSs) in full-arch implant-supported prosthodontics. A standardized maxillary model with six implants was used to reproduce the clinical challenge of screw-retained full-arch frameworks. By employing a single reference model, we eliminated inter-patient anatomical variability, ensuring that deviations observed were attributable solely to scanner performance.

### 2.2. Reference Model and Ground-Truth Dataset

The experimental model consisted of an extra-hard dental stone maxillary cast fitted with a silicone gingival mask. Six multi-unit analogs (Ø 4.8 mm, regular platform MegaGen^®^ Multi-Unit System, MegaGen Implant Co., Ltd., Daegu, Republic of Korea) were used in the maxillary model, and were positioned to support a screw-retained full-arch prosthesis: two anteriorly in the central/lateral incisor region, two premolars, and two molars. The inter-implant distance ranged from approximately 10–14 mm in the anterior region to 20–22 mm posteriorly, reproducing typical full-arch spacing.

**Figure 1:**
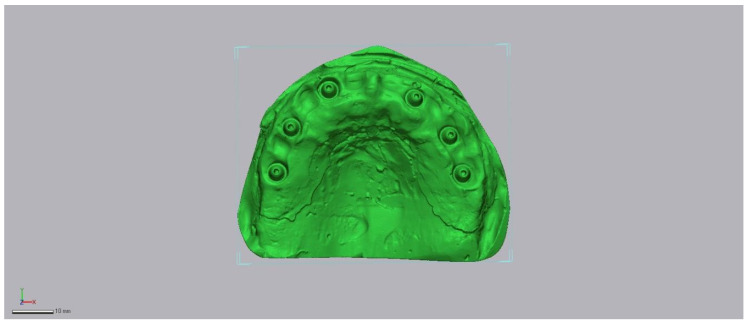
Reference laboratory scan of the maxillary model with six implants (DD Argus M2 HD). Scale bar = 10 mm.

The model was digitized using a DD Argus M2 HD laboratory scanner (DOF Inc., Seoul, Republic of Korea). Calibration was performed according to the manufacturer’s protocol, achieving <10 μm accuracy. The manufacturer-stated accuracy was verified using calibration logs and traceable measurement artifacts according to ISO 12836:2015 [19], ensuring reliability of the reference model. A uniform layer of anti-glare scanning spray (particle size < 3 µm) was applied to optimize surface capture and minimize reflectivity. The resulting STL (Standard Tessellation Language) dataset served as the reference (Figure 1).

### 2.3. Test Scanners and Native Software

Three commonly used IOSs were selected for comparison:

• TRIOS Core (3Shape A/S, Copenhagen, Denmark)—10 scans, processed with 3Shape software (version 24.1).
• Medit i700 (Medit Corp., Seoul, Republic of Korea)—10 scans, processed with Medit Link software (version 3.4.5).
• Primescan (Dentsply Sirona, Bensheim, Germany)—10 scans, processed with CEREC software (version 5.3.2).

**Figure 2:**
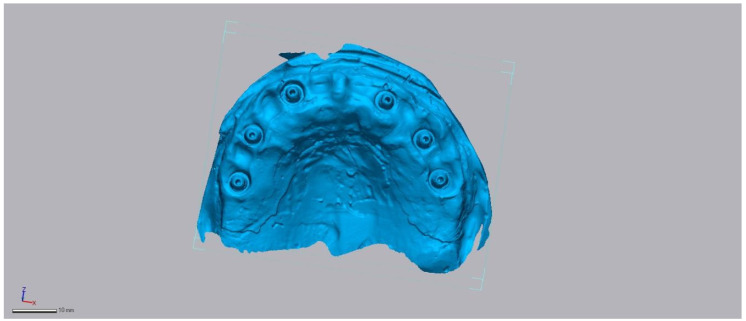
Medit i700 intraoral scan example (raw STL export, no mesh edits).

**Figure 3:**
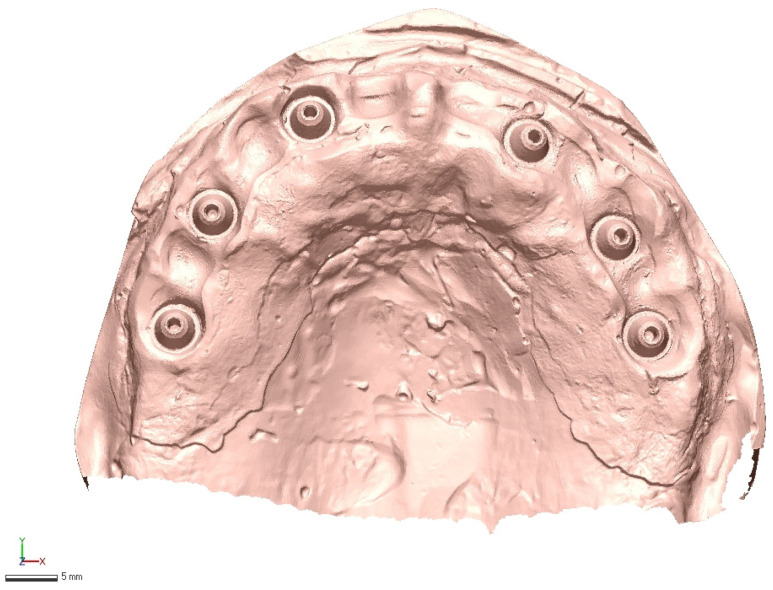
3Sape intraoral scan example (raw STL export, no mesh edits).

**Figure 4:**
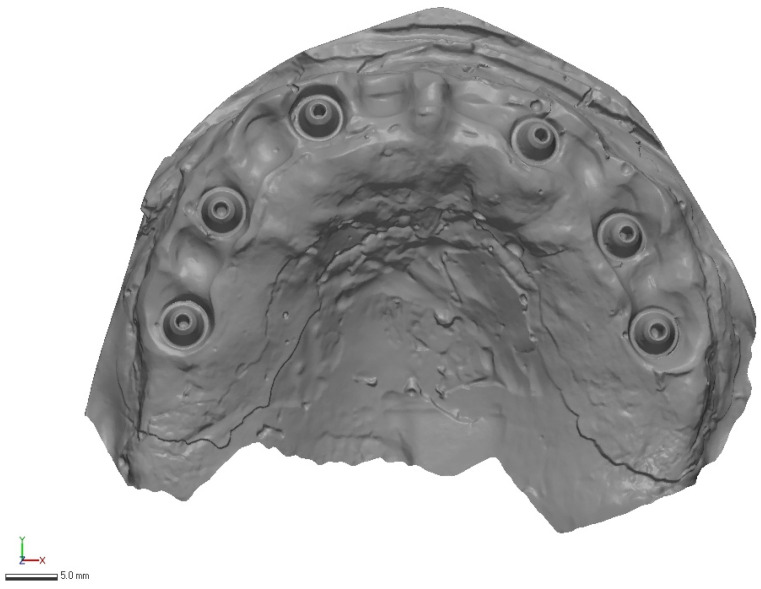
Primescan (raw STL export, no mesh edits).

In total, 30 IOS scans were generated, each exported in STL format with no mesh modification (no smoothing, decimation, or repair) before analysis. Representative raw acquisitions are shown in Figure 2, Figure 3 and Figure 4.

### 2.4. Operators and Environment

All scans were acquired by two prosthodontists with over 8 years of clinical experience each (>300 IOS clinical cases performed). To minimize operator-related variability, both followed identical protocols and were trained in advance on the test model. Scanning was carried out in a standardized laboratory environment: 22 ± 1 °C, uniform overhead illumination, and the model stabilized on a matte, non-reflective platform.

Two prosthodontists with at least eight years of clinical experience each (over 300 IOS clinical cases) performed all scans. To minimize operator bias, both operators followed the same standardized scanning trajectory and performed an equal number of scans per device (five each). The scanning order for each device was randomized to control for sequence or learning effects. Operator influence was preliminarily assessed and found to be smaller than inter-scanner variability; however, the limited number of operators did not justify a nested or mixed model, and this is acknowledged as a limitation in the Discussion.

All scans were performed by two licensed prosthodontists affiliated with the Department of Prostheses Technology and Dental Materials, Victor Babeș University of Medicine and Pharmacy, Timișoara, Romania. Each operator has more than eight years of clinical experience and routinely performs intraoral scanning in implant-supported prosthodontics.

### 2.5. Scanning Protocol

Each complete acquisition included three sequential steps:

1. Capture of the gingival mask and peri-implant soft tissues;
2. Recording of the six multi-unit analogs;
3. A second pass with scan bodies attached, enabling three-dimensional capture of implant axes and positions.

Manufacturer-recommended scanning strategies were strictly followed. TRIOS Core and Medit i700 employed a pendular sweeping motion with frame overlap, while Primescan used a linear trajectory.

All scans were performed using default manufacturer-recommended settings. Acquisition speed for TRIOS Core was approximately 20–25 mm/s with adaptive exposure control enabled, while Medit i700 scans were carried out at 18–22 mm/s using automated brightness adjustment. Primescan acquisitions proceeded at 15–20 mm/s with dynamic exposure compensation. Titanium Grade 5 scan bodies with matte surface finish (Dess^®^, Barcelona, Spain) were attached during the implant recording stage to ensure stable geometry capture. The model surface was dried prior to scanning to prevent reflection artifacts, and small stitching errors were corrected by selective rescanning of the affected area.

### 2.6. Region of Interest and Alignment Strategy

**Figure 5:**
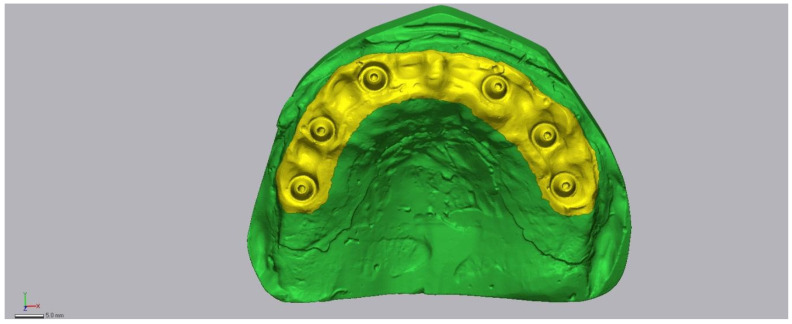
Isolated region of interest (ROI) used for metrology in Geomagic Control X.

**Figure 6:**
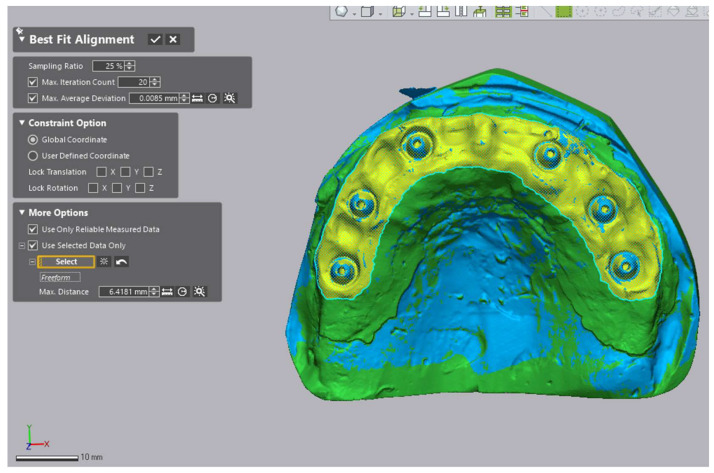
Two-step registration workflow: initial alignment followed by ROI-restricted best-fit (ICP).

All meshes were imported into Geomagic Control X (v16.0.2.16496; 3D Systems, Rock Hill, SC, USA). A peri-implant Region of Interest (ROI) encompassing the scan bodies, adjacent soft tissue, and peri-implant zones was isolated and saved as a template (Figure 5). Each dataset was aligned to the reference in two steps:

(1) initial rough alignment, followed by
(2) an iterative closest point (ICP) best-fit restricted to the predefined peri-implant Region of Interest (ROI) (Figure 6).

The ROI included the scan bodies and adjacent peri-implant soft tissues. We recognize that aligning and measuring within the same ROI may partially minimize deviations, a limitation described in surface-based metrology studies. To ensure comparability, the ROI template was consistently applied across all scanners. Future studies should explore alignment on independent features or full-arch registration with separate evaluation ROIs to further assess sensitivity to alignment constraints.

### 2.7. Accuracy Assessment

Accuracy was defined as:

• Trueness: mean absolute surface deviation (μm) between test and reference meshes;
• Precision: variability of trueness across 10 replicates (standard deviation).

**Figure 7:**
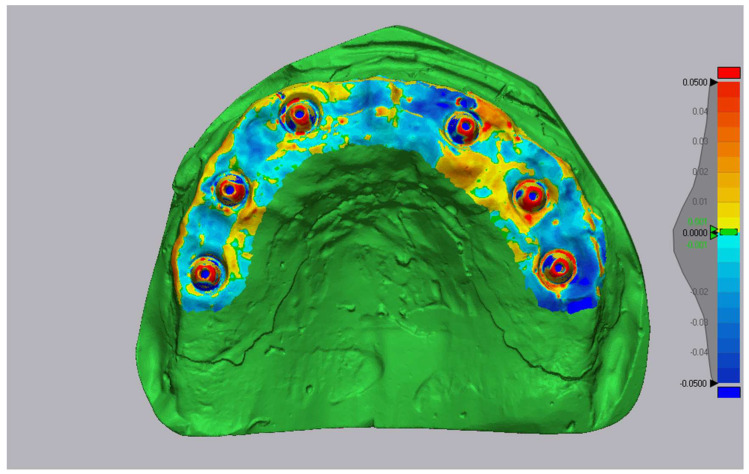
Three-dimensional comparison map—Medit i700 vs. reference (Tolerance ± 0.05 mm (green ± 50 µm)).

**Figure 8:**
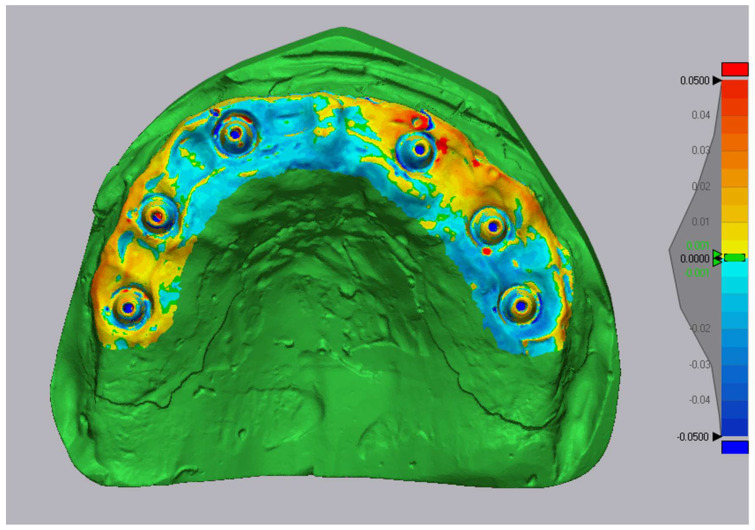
Three-dimensional comparison map-Primescan vs. reference (Tolerance ± 0.05 mm (green ± 50 µm)).

**Figure 9:**
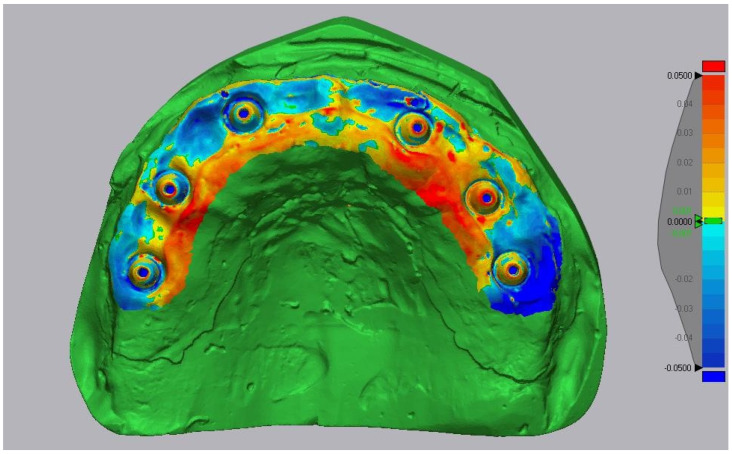
Three-dimensional comparison map-TRIOS Core vs. reference (Tolerance ± 0.05 mm (green ± 50 µm)).

Deviation color maps were generated with a tolerance of ±0.05 mm. Green indicated surfaces within tolerance (±50 μm), red represented outward deviations, and blue inward deviations. The previous label ‘±1 μm’ was a typographical error and has been corrected. Representative examples are shown in Figure 7, Figure 8 and Figure 9.

This surface-based deviation approach using iterative closest point alignment has been widely applied in digital implant impression accuracy studies [20,21].

### 2.8. Quality Control and Sample Size Rationale

Scans were excluded if they presented incomplete geometry, stitching errors, or uncorrectable misalignments. No scans were excluded in the present study, ensuring a complete dataset for all scanners. The replicate count (*n* = 10 per scanner) followed previous metrology studies [22], which recommend ≥8–10 replicates to detect mean differences of 10–20 μm with >80% statistical power. A priori power analysis (f = 0.40, α = 0.05, power = 0.80) was based on an expected standard deviation of 10–15 µm, consistent with previous full-arch scanner accuracy studies. This indicated that ten replicates per group were sufficient to detect 10–20 µm mean differences. The underlying assumptions and variance structure are now explicitly reported for transparency.

### 2.9. Statistical Analysis

Data analysis was performed in MedCalc (v20.218; MedCalc Software Ltd., Ostend, Belgium). Normality was tested with the Kolmogorov–Smirnov test, and variance homogeneity with Levene’s test. Differences in trueness between scanners were assessed using one-way ANOVA (α = 0.05). When significant, Tukey’s honestly significant difference (HSD) test was applied for pairwise comparisons. Results are reported as mean ± SD (μm) with 95% confidence intervals. Effect sizes (η^2^, ω^2^) were calculated. Visualizations included box plots with replicate overlays.

## 3. Results

### 3.1. Accuracy Values

The comparative evaluation of the three intraoral scanners revealed distinct performance patterns. Primescan exhibited the lowest mean deviation from the reference model (202.76 µm), followed very closely by TRIOS Core (204.21 µm). The difference of approximately 1–2 µm is statistically insignificant and clinically negligible; therefore, both scanners can be regarded as comparable in trueness. The 3Shape TRIOS Core demonstrated a nearly identical mean value (204.21 μm), with only about 1 μm difference compared to Primescan-an amount that can be regarded as clinically negligible. In contrast, the Medit i700 produced higher deviations (221.05 μm), with a difference of approximately 16–18 μm compared to the other two devices.

When variability across repeated scans was examined, the TRIOS Core stood out with the smallest standard deviation (2.61 μm) and the narrowest 95% confidence interval, reflecting excellent reproducibility. Primescan, while accurate on average, showed greater variability between replicates (SD = 13.89 μm), whereas Medit i700 displayed intermediate reproducibility (SD = 6.28 μm). These findings suggest that although both Primescan and TRIOS Core perform similarly in terms of trueness, TRIOS Core may provide more consistent results across repeated clinical applications.

**Figure 10:**
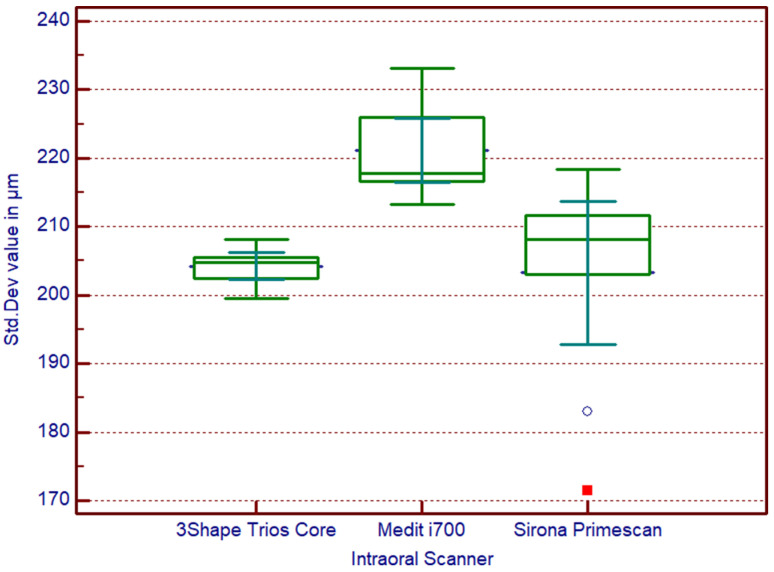
Distribution of trueness values for the three intraoral scanners. Boxplots show the tight clustering of TRIOS Core measurements, the wider spread of Primescan, and the consistently higher deviations of Medit i700. Tolerance band = ±0.05 mm (green = within tolerance, red = outward deviation, blue = inward deviation).

The distribution of values is illustrated in Figure 10, where the boxplots demonstrate the tight clustering of TRIOS Core measurements, the wider spread of Primescan, and the consistently higher deviations associated with Medit i700.

### 3.2. Statistical Analysis Results

The inferential statistics supported the descriptive findings. Table 1 summarizes the mean trueness values and variability for each scanner. Given the variance heterogeneity (*p* = 0.014), Welch’s ANOVA was also performed and confirmed identical significance patterns (*p* < 0.001), ensuring robustness of the inference despite unequal variances. Because Levene’s test (Table 2) indicated unequal variances (*p* = 0.014), Welch’s ANOVA was additionally performed to verify robustness. The significance pattern remained unchanged (*p* < 0.001), confirming the stability of the results under variance-heterogeneous conditions. Despite this, the balanced study design (*n* = 10 per group) allowed valid comparisons.

One-way ANOVA (Table 3) showed that scanner type had a highly significant effect on trueness (F(2,27) = 11.39, *p* < 0.001), with a large effect size (η^2^ = 0.46), highlighting the strong influence of device choice on accuracy. Post hoc analysis using Tukey’s HSD (Table 4) further clarified these relationships: Medit i700 differed significantly from both Primescan and TRIOS Core (*p* < 0.05), confirming its lower accuracy. In contrast, no significant difference was found between Primescan and TRIOS Core (*p* > 0.05), corroborating the negligible discrepancy between their mean values.

**Table 1:** Mean trueness values (μm) for intraoral scanners (mean ± SD, *n* = 10 per scanner).

Scanner	Mean (μm)	SD (μm)	95% CI
Medit i700	221.05	6.28	216.9–225.2
Sirona Primescan	202.76	13.89	193.1–212.4
3shape TRIOS Core	204.21	2.61	202.4–206.0

**Table 2:** Levene’s test for homogeneity of variances.

Statistic	df1	df2	*p*-Value
**4.990**	2	27	0.014

### 3.3. Experimental Conclusions

Taken together, the results demonstrate that:

• Primescan offers the best trueness on average, but with higher variability between scans.
• TRIOS Core provides comparable trueness while demonstrating the most consistent performance across repeated scans.
• Medit i700, although falling within ranges considered clinically acceptable, showed significantly greater deviations, which could become critical in demanding full-arch, screw-retained protocols where passive fit is essential.

These findings underline that scanner choice should not be based solely on average accuracy values, but also on reproducibility, especially when treating complex implant-supported prosthodontic cases.

## 4. Discussion

This in vitro study demonstrated measurable accuracy differences among three intraoral scanners used for full-arch implant impressions. Sirona Primescan exhibited the highest trueness, closely followed by 3Shape TRIOS Core, while Medit i700 showed significantly greater deviations. TRIOS Core also demonstrated superior reproducibility across repeated scans. These variations are relevant because accuracy plays a critical role in achieving passive fit in screw-retained full-arch prostheses. Such variability in full-arch and edentulous/implant scenarios has been consistently reported in prior 3D metrology studies [23,24,25,26].

No predefined clinical threshold was applied in this study. Although scanner trueness and precision contribute to clinical acceptability, no universally defined numerical threshold exists for an acceptable misfit in full-arch implant prosthodontics. Therefore, the present results should be interpreted primarily as comparative metrological outcomes rather than direct indicators of clinical performance. Clinical acceptability depends not only on accuracy values but also on reproducibility, implant distribution, prosthetic design, and intraoral factors that cannot be replicated in vitro; accordingly, accuracy metrics alone cannot determine the overall clinical suitability of an intraoral scanner [13,20,23,24,27,28,29]. The deviations recorded here (>200 µm) reflect the stricter surface-based methodology, which assesses extended peri-implant regions and therefore yields higher values than point-based measurements typically used in clinical studies [23,25,30].

Methodological differences also explain the discrepancy between our values and those reported for analog impressions. Papazoglou et al. documented discrepancies of 19.3–47.3 µm in complete-arch analog impressions [30], markedly lower than the surface-based deviations identified in the present study. Nonetheless, the ranking of scanners observed here remained stable and clinically meaningful. Consistent with other in vitro reports [6,13,20,31], scanners exhibiting higher trueness are associated with a lower risk of framework misfit and fewer mechanical or biological complications in long-span rehabilitations [12,13,14,15,16]. The scanning procedures were carried out by two experienced prosthodontists regularly involved in full-arch implant rehabilitation, ensuring that the acquisition protocols reflected current clinical practice. Therefore, although the study was conducted in vitro, the workflow closely replicates routine clinical implementation of intraoral scanning for screw-retained complete-arch prostheses.

Although conducted in vitro, scanning was performed by two experienced prosthodontists routinely involved in full-arch implant rehabilitation, ensuring that acquisition protocols reflected current clinical practice. The small number of scans per operator, however, did not permit formal inter-operator reliability testing, and operator variability must be acknowledged as a limitation.

From a clinical perspective, the impression stage must achieve accuracy levels exceeding the expected framework tolerance, since additional discrepancies arise during model fabrication, milling, and clinical try-in. Systematic reviews confirm that full-arch IOS trueness ranges from single-digit to several hundred microns, underscoring the importance of minimizing deviation whenever possible [21,28,32,33]. Evidence also shows that hybrid workflows -such as splinted analog impressions or photogrammetry-often achieve lower deviations than IOSs alone, supporting their use in high-precision full-arch cases [34,35].

The practical implications of these findings depend on the restorative scenario. Primescan may be preferable where minimizing trueness bias is essential, such as immediate loading protocols. TRIOS Core offers highly consistent performance, making it suitable for workflows requiring repeated scans or long-term monitoring. Medit i700, although less accurate, remained reproducible and may be adequate for less demanding indications or cost-constrained settings. Emerging technologies such as photogrammetry and stereophotogrammetry can further enhance implant pose accuracy and may complement IOSs in complex full-arch rehabilitations [21,31,32].

This study has limitations, including its in vitro design, single model, controlled laboratory environment, use of one ROI for alignment and evaluation, and the inclusion of only three scanners. The surface-based accuracy assessment may overestimate deviations compared with point-based methods, and the absence of alternative digital workflows (e.g., photogrammetry) limits the breadth of comparison. Future research should include in vivo evaluations, larger operator pools, mixed-effects statistical models, and hybrid digital protocols to determine how scanner accuracy translates to long-term clinical success.

Future research should build upon these findings through well-designed in vivo studies that evaluate scanner performance under real clinical conditions, where variables such as saliva, patient movement, soft-tissue dynamics, and intraoral lighting may further influence accuracy. Comparative investigations involving a wider range of implant distributions, edentulous arch anatomies, and prosthetic designs are needed to understand how scanner performance scales with increasing case complexity. Additionally, hybrid digital workflows combining intraoral scanning with photogrammetry or stereophotogrammetry should be explored systematically, as these approaches may offer improved implant pose accuracy in full-arch rehabilitations. Expanding operator pools and using mixed-effects statistical models will be essential for quantifying operator-related variance. Finally, long-term clinical studies should assess whether measured deviations correlate with prosthetic fit, mechanical complication rates, peri-implant tissue stability, and overall restoration longevity.

## 5. Conclusions

This in vitro study compared the trueness and precision of three commonly used intraoral scanners in a standardized full-arch implant model. Sirona Primescan and 3Shape TRIOS Core demonstrated comparable trueness, while TRIOS Core showed superior reproducibility across repeated scans. Medit i700 presented significantly higher deviations, though still within ranges considered manageable in controlled digital workflows. These results highlight measurable differences in scanner performance under standardized laboratory conditions and provide evidence to guide device selection in full-arch digital implant prosthodontics. Further in vivo studies are required to validate these findings in clinical environments.

## Figures and Tables

**Table 3:** One-way ANOVA for trueness values among scanners.

Source of Variation	Sum of Squares	df	Mean Square	F	*p*-Value
**Between groups**	2020.69	2	1010.34	11.393	<0.001
**Within groups**	2394.49	27	88.68		
**Total**	4415.17	29			

**Table 4:** Tukey’s HSD post hoc pairwise comparisons (α = 0.05).

Scanner	Mean (μm)	Significant vs.
**3shape Trios Core**	204.21	Medit i700
**Medit i700**	221.05	(Primescan, TRIOS Core)
**Sirona Primescan**	202.76	(Medit i700)

Note: Medit i700 differs from both Primescan and TRIOS Core (*p* < 0.05). Primescan vs. TRIOS Core, not significant (*p* > 0.05).

## Data Availability

The original contributions presented in this study are included in the article. Further inquiries can be directed to the corresponding authors.

## Abbreviations

The following abbreviations are used in this manuscript:

IOS	intraoral scanner
ROI	region of interest
ICP	iterative closest point
SD	standard deviation
ANOVA	analysis of variance
HSD	honestly significant difference
CI	confidence interval
